# Simple MRI score aids prediction of dementia in cerebral small vessel disease

**DOI:** 10.1212/WNL.0000000000009141

**Published:** 2020-03-24

**Authors:** Ali Amin Al Olama, James M.S. Wason, Anil M. Tuladhar, Esther M.C. van Leijsen, Marisa Koini, Edith Hofer, Robin G. Morris, Reinhold Schmidt, Frank-Erik de Leeuw, Hugh S. Markus

**Affiliations:** From the Stroke Research Group (A.A.A.O., H.S.M.), Clinical Neurosciences, University of Cambridge; MRC Biostatistics Unit (J.M.S.W.), Institute of Public Health, Cambridge; Institute of Health and Society (J.M.S.W.), Newcastle University, UK; Department of Neurology (A.M.T., E.M.C.v.L., F.-E.d.L.), Radboud University Nijmegen Medical Centre, Donders Institute for Brain, Cognition and Behaviour, Centre for Medical Neuroscience, Nijmegen, the Netherlands; Division of Neurogeriatrics (M.K., E.H., R.S.), Department of Neurology, Medical University of Graz; Institute for Medical Informatics (E.H.), Statistics and Documentation, Medical University of Graz, Austria; and Department of Psychology (R.G.M.), King's College, Institute of Psychiatry, Psychology and Neuroscience, London, UK.

## Abstract

**Objective:**

To determine whether a simple small vessel disease (SVD) score, which uses information available on rapid visual assessment of clinical MRI scans, predicts risk of cognitive decline and dementia, above that provided by simple clinical measures.

**Methods:**

Three prospective longitudinal cohort studies (SCANS [St George's Cognition and Neuroimaging in Stroke], RUN DMC [Radboud University Nijmegen Diffusion Imaging and Magnetic Resonance Imaging Cohort], and the ASPS [Austrian Stroke Prevention Study]), which covered a range of SVD severity from mild and asymptomatic to severe and symptomatic, were included. In all studies, MRI was performed at baseline, cognitive tests repeated during follow-up, and progression to dementia recorded prospectively. Outcome measures were cognitive decline and onset of dementia during follow-up. We determined whether the SVD score predicted risk of cognitive decline and future dementia. We also determined whether using the score to select a group of patients with more severe disease would reduce sample sizes for clinical intervention trials.

**Results:**

In a pooled analysis of all 3 cohorts, the score improved prediction of dementia (area under the curve [AUC], 0.85; 95% confidence interval [CI], 0.81–0.89) compared with that from clinical risk factors alone (AUC, 0.76; 95% CI, 0.71–0.81). Predictive performance was higher in patients with more severe SVD. Power calculations showed selecting patients with a higher score reduced sample sizes required for hypothetical clinical trials by 40%–66% depending on the outcome measure used.

**Conclusions:**

A simple SVD score, easily obtainable from clinical MRI scans and therefore applicable in routine clinical practice, aided prediction of future dementia risk.

MRI features of cerebral small vessel disease (SVD) are associated with increased dementia risk,^1^ but predicting whether an individual patient with SVD will progress to dementia has not been possible.

One possible approach is to use information available on clinical MRI scans. An SVD severity score has been proposed^2^ that is easily applicable in clinic and combines several MRI markers including lacunar infarcts and white matter hyperintensities (WMH), as well as cerebral microbleeds (CMB) and perivascular spaces (PVS), all of which can be assessed simply and rapidly by visual inspection of clinical MRI scans. This score has been shown to associate with cognitive impairment in patients with symptomatic lacunar stroke and in community populations^3,4^ but such cross-sectional studies cannot determine whether it predicts future risk of dementia and cognitive decline. To address this, longitudinal studies are required with several years of follow-up.

The aim of this study was to determine whether a simple MRI score can predict dementia. We examined its performance in 3 prospective longitudinal cohorts, which included patients with SVD ranging from mild and asymptomatic to severe and symptomatic. The MRI score^2^ divides the individual MRI markers in a binary fashion (i.e., presence or absence). Therefore we further assessed the effect of including more detailed information into the scores; for example, by grading the number of lacunar infarcts and the severity of WMH on an ordinal scale. We also determined whether using the score to preselect patients with a higher chance of developing dementia would reduce sample sizes in trials of agents to reduce cognitive decline in SVD.

## Methods

### Cohorts studied

Three longitudinal studies were included. St George's Cognition and Neuroimaging in Stroke (SCANS) included moderate to severe symptomatic SVD, the Radboud University Nijmegen Diffusion Imaging and Magnetic Resonance Imaging Cohort (RUN DMC) included all grades of symptomatic SVD, and the Austrian Stroke Prevention Study (ASPS) covered a community population with mild MRI evidence of SVD.

### Standard protocol approvals, registrations, and patient consents

Local ethical approval was obtained for each study and each participant gave written informed consent.

### SCANS

Patients with SVD were screened and invited between 2007 and 2010 from 3 stroke services in South London, United Kingdom.^5,6^ SVD was defined as a clinical lacunar stroke syndrome as well as confluent WMH on MRI. Participants were excluded if they presented with any cause of stroke mechanism other than SVD or a clinical diagnosis of dementia. Study participants (n = 121) had multimodal MRI and cognitive tests performed at baseline and at years 1, 2, and 3 as well as follow-up for dementia to year 5. Ninety-nine participants returned at 1 or more time points for MRI measures. For this analysis, we used MRI data from the baseline visit, cognitive data detecting change from baseline to year 3, and data collected on progression to dementia over a 5-year follow-up. MRI sequences performed on a 1.5T Signa HDxt MRI system (General Electric, Milwaukee, WI) included whole-brain T1-weighted, fluid-attenuated inversion recovery (FLAIR), and gradient echo. Follow-up data on dementia incidence was available for all 121 participants.

### RUN DMC

RUN DMC is a prospective study of symptomatic SVD. Full methodologic details have been published.^7^ A total of 503 individuals without dementia with radiologic SVD defined as the presence of 1 or more lacunes or WMH were recruited in 2006. Study participants were assessed at baseline and at follow-up in 2011 and in 2015. Assessment included cognitive, MRI, and clinical assessments. We used the baseline MRI data from the 2006 visit and follow-up data on cognition and incidence of dementia from the 2011 and 2015 visits. Follow-up data on dementia incidence were available for 501 participants. MRI sequences, performed on a 1.5T Magnetom scanner (Siemens, Erlangen, Germany), included whole-brain T1-weighted, FLAIR, and gradient echo.

### ASPS

ASPS is a community-based cohort study in participants without a history or signs of stroke or dementia in Graz, Austria. Full methodologic details have been published.^8,9^ The data used for our study consisted of 2 cohorts: 871 participants from the elderly ASPS cohort and 347 from the ASPS-family study.^10^ A total of 541 participants (194 from ASPS and 347 from the family cohort) had complete MRI data available, which allowed construction of the SVD score, and they were included in the baseline analysis. A total of 193 of 194 individuals from the elderly cohort were followed at 3 time points for a maximum of 11 years (median follow-up of 3 years) and data on dementia incidence were available in all 193. Of the 347 individuals from the family cohort, 126 were followed at 2 time points for a maximum of 8 years (median follow-up of 2 years), and dementia data were available in all 126. There was no difference between those dropping out of the family cohort in median (interquartile range [IQR]) age (drop out 68 [55–74] years, remained in 67 [62–72] years; *p* = 0.696) or in median (IQR) years of education (drop out 10 [10–13] years, remained in 10 [10–13] years; *p* = 0.128), but those dropping out were more likely to be female (64.2% vs 52.7%; *p* = 0.035). There was no overlap between the 2 cohorts and data were combined for analysis. MRI scans were performed on 1.5T scanners (Gyroscan S 15 and ACS; Philips, Eindhoven, the Netherlands) for elderly cohort participants and included proton density, T2-weighted sequences, and T1-weighted images. Participants in the family cohort were examined by a 3T whole body scanner (TimTrio; Siemens Healthcare, Erlangen, Germany).

### Cognition assessments and construction of cognitive indices

In each cohort, a battery of neuropsychological tests was administered and cognitive index scores constructed to allow assessment of global cognition (a measure of overall performance across all tasks) and 2 specific domains particularly affected in SVD^11^: processing speed (PS) and executive function (EF). Index scores for each cognitive domain were calculated by averaging the component global or cognitive measures. The different tests used to construct the index scores are shown in table 1.

**Table 1:**
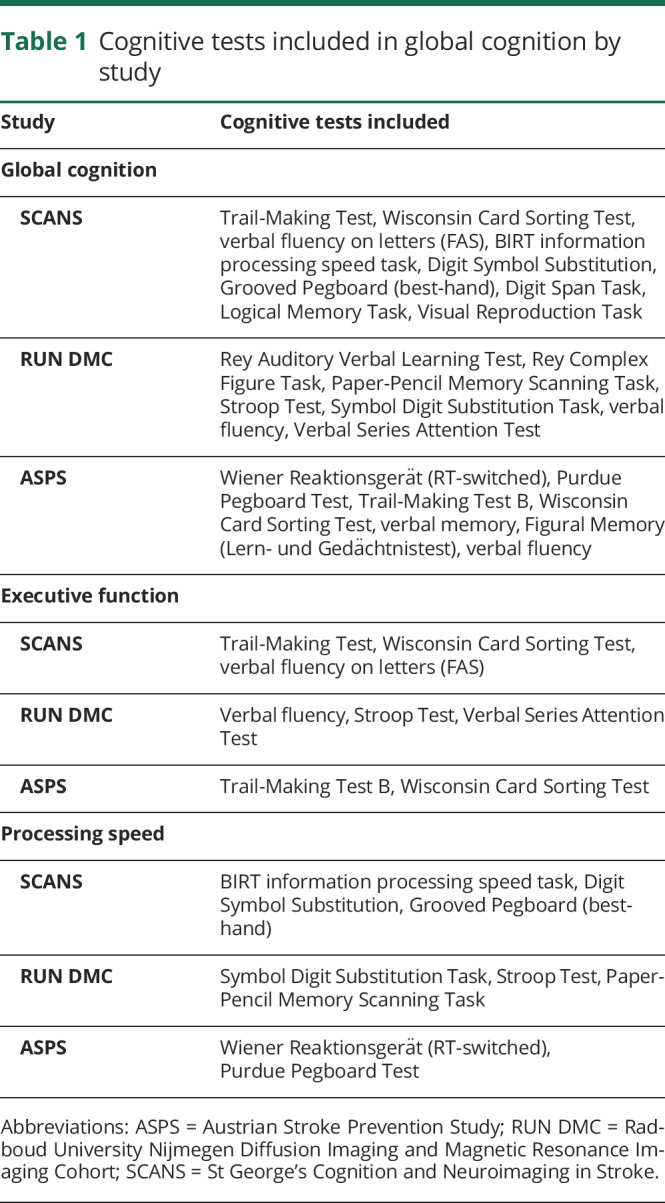
Cognitive tests included in global cognition by study

In SCANS,^6^ the cognitive measures were converted into *Z* scores based on published normative data, using age at baseline for scaling purposes, and then aggregated into the index scores. For RUN DMC^7,12^ and ASPS^8,9^ data, the measures were converted into *Z* scores based on the baseline mean and SDs of the raw scores before aggregation.

### Conversion to dementia

In all cohorts, participants developing dementia during follow-up were identified. In SCANS, dementia was diagnosed using the DSM-V definition^13^ of major neurocognitive disorder and identified as described previously.^6^ In RUN DMC, dementia diagnosis was based on DSM-IV criteria as described previously^12^; probable Alzheimer disease (AD) was based on National Institute on Aging–Alzheimer’s Association criteria and vascular dementia on National Institute of Neurological Disorders and Stroke–Association Internationale pour la Recherche en l’Enseignement en Neurosciences criteria.^14^ In ASPS, dementia was defined by a Mini-Mental State Examination score <24 points at follow-up for the majority of participants (except 3 participants in whom the diagnosis was recorded in the medical records), and diagnosis of dementia subtype (Alzheimer vs vascular) was not available.

Analysis was performed for all-cause dementia and then repeated for only vascular dementia cases. For this latter analysis, only vascular dementia cases were included for SCANS and RUN DMC but all cases were included from ASPS.

### Generation of total SVD score

A total SVD score was generated as described previously.^2^ One point was given for the presence of each of any lacunar infarct, WMH, and CMB. Because PVS data were not available in RUN DMC, and no correlation had been shown between PVS counts and cognition previously in SCANS,^15^ a simple SVD score without PVS was generated, and therefore the simple SVD score had a range from 0 to 3. However, to assess the additional predictive value of PVS, an additional sensitivity analysis was performed to investigate the impact of excluding PVS information, in which an SVD score including PVS (0–4) was compared with a simple SVD score excluding PVS (0–3) in the SCANS and ASPS datasets.

The simple SVD score was also modified to determine whether including more information on the severity of MRI features affects its performance. In this modified score, WMH were graded from 0 to 3 using the Fazekas scale,^16^ and the number of lacunar infarcts was graded from 0 to 3 (0 = none, 1 = 1 to 2, 2 = 3 to 5, 3 = >5). CMB were still graded as absent (0) or present.^1^ The amended SVD score therefore had a range from 0 to 7. The details of the 2 SVD scores are shown in table 2.

**Table 2:**
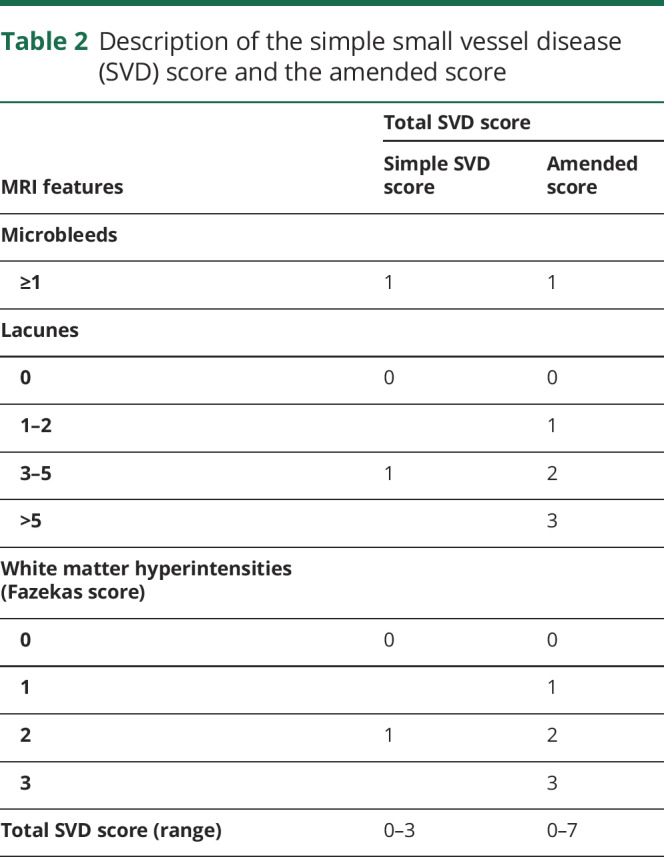
Description of the simple small vessel disease (SVD) score and the amended score

### Statistical methods

Analyses were performed for both the simple SVD score and the amended SVD score. First the association of simple SVD scores with global cognition, EF, and PS was determined using linear regression at baseline. Akaike information criterion (AIC) was estimated to compare the relative fit of statistical models. We then determined whether the baseline scores (simple and amended scores) predicted future cognitive decline and progression to dementia. To determine whether the scores predicted dementia, logistic regression was performed and the classification ability of the scores for predicting dementia assessed. The area under the curve (AUC) was estimated for a model including age, sex, and education years, and this was compared with a similar model in which the SVD score was added.

Slopes of change in cognition were estimated using a linear mixed effect (LME) model utilizing all time points with cognitive information. We then performed analysis of variance to compare the slopes among simple SVD score groups by using slopes on an individual level for each study. This was performed for each study and a *p* value was presented. All models were adjusted for age, sex, and years of education.

A sample size calculation was performed for a hypothetical trial to determine the effect on power of selecting patients with severe SVD identified by a simple SVD score ≥2. We calculated minimum sample sizes per arm with 80% power to detect a range of treatment effect from 10%, 15%, 20%, 25%, and 30% in the intervention group. Sample sizes were calculated for 2 outcomes: (1) cognitive change and (2) conversion to dementia. Sample size estimation was carried out for cognitive indexes using the longpower package in R and LME model was used to estimate the intercept, slope variances, and residual variances. For the binary endpoint of progression to dementia, a Cox regression model was used with 5 years follow-up. This is the longest duration follow-up likely to be relevant for a clinical trial. The powerSurvEpi package in R was used for sample size calculation. All statistical analyses were carried out in R version 3.3.1.

### Data availability

Requests for data from ASPS, SCANS, and RUN DMC should be made to the individual studies (reinhold.schmidt@medunigraz.at [ASPS], hsm32@medschl.cam.ac.uk[SCANS], frankerik.deleeuw@radboudumc.nl[RUN DMC]).

## Results

Table 3 summarizes the demographics and risk factors of the 3 cohorts. SCANS had participants with the most severe SVD and ASPS participants with the least severe SVD as reflected in the proportion of cases with a simple SVD score ≥2: 65% in SCANS, 21% in RUN DMC, and 9% in ASPS.

**Table 3:**
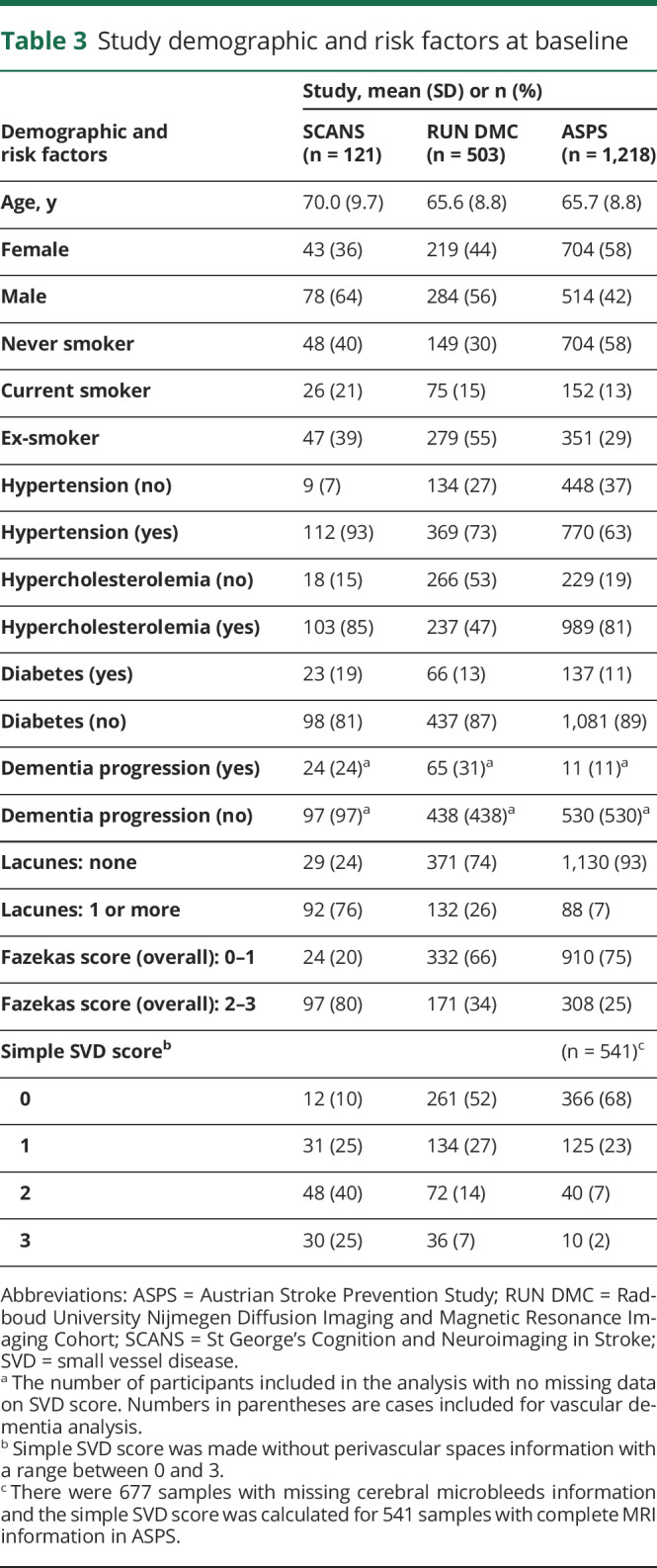
Study demographic and risk factors at baseline

### Associations with baseline cognition

There were negative associations between the simple SVD score and baseline cognitive indices. The associations were statistically significant for the global cognition index and all cognitive domain indices except for EF in RUN DMC and ASPS (table e-1, doi.org/10.17863/CAM.48785). There was a slight improvement in how well the model fitted (AIC difference >2) when we used the amended score in SCANS, but the fit was similar in ASPS and RUN DMC (AIC difference <2).

### Prediction of dementia

**Figure 1:**
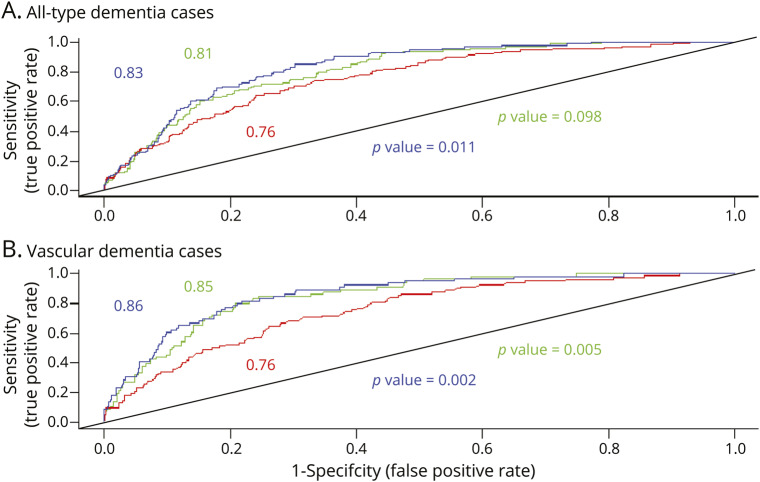
Receiver operating characteristic (ROC) curves illustrate the improved prediction of dementia when the simple MRI score is added to a model adjusted for age, sex, and education Results are shown for a model without the MRI score (red line), after adding a simple MRI score (green line), and after adding an amended MRI score (blue line). *p* Value shows the statistical difference among ROC curves (blue: simple small vessel disease [SVD] score vs model without score; green: amended SVD score vs model without score).

Adding the simple SVD score to a model including age, sex, and education years improved prediction as evidenced by an increased AUC. In an analysis of the pooled datasets, the AUC improved from 0.76 (95% confidence interval [CI], 0.72–0.80) to 0.81 (95% CI, 0.77–0.85) in a model with the simple SVD score (*p* = 0.098) and to 0.83 (95% CI, 0.80–0.87) with the amended score (*p* = 0.011). When we used vascular dementia cases as the outcome, the prediction became slightly stronger with AUC = 0.85 (0.81–89) and 0.86 (0.82–0.90) in a model with the simple score (*p* = 0.005) and with the amended score (*p* = 0.002), respectively. Using the amended score appeared to increase the AUC in SCANS (0.71 vs 0.74) more than RUN DMC (0.81 vs 0.82) and ASPS (0.83 vs 0.84) (table 4 and figure 1).

**Table 4:**
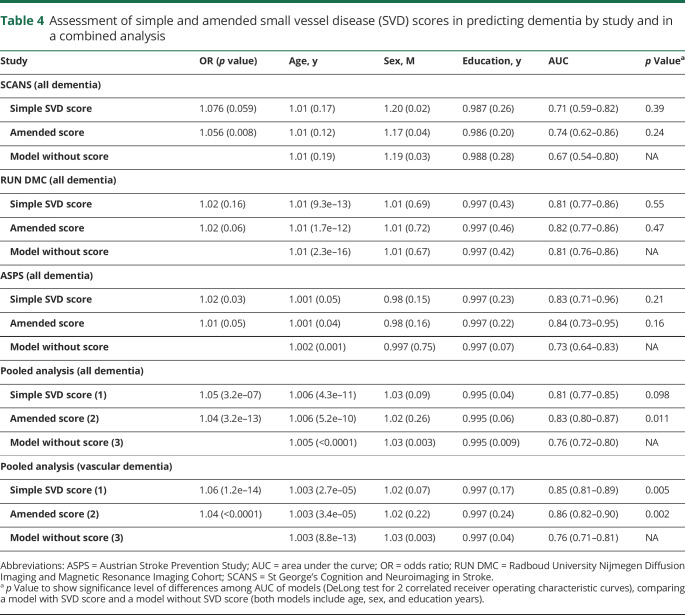
Assessment of simple and amended small vessel disease (SVD) scores in predicting dementia by study and in a combined analysis

A sensitivity analysis, in which the original score^10^ including PVS was compared to the simple score without PVS, showed no significant difference in AUCs (0.69 vs 0.71, *p* value = 0.35) and demonstrated that adding PVS did not improve model prediction (table e-2, doi.org/10.17863/CAM.48785).

We also compared the performance of the score with a model that also included vascular risk factors (hypertension, hypercholesterolemia, diabetes mellitus, and smoking) in addition to age, sex, and education. Including these vascular risk factors slightly improved prediction of dementia from C = 0.76 to 0.80. The score still resulted in higher prediction (0.82 for the simple score and 0.84 for the amended score; amended score vs model without MRI score *p* = 0.03) but the additional prediction provided was smaller.

### Prediction of cognitive decline

**Figure 2:**
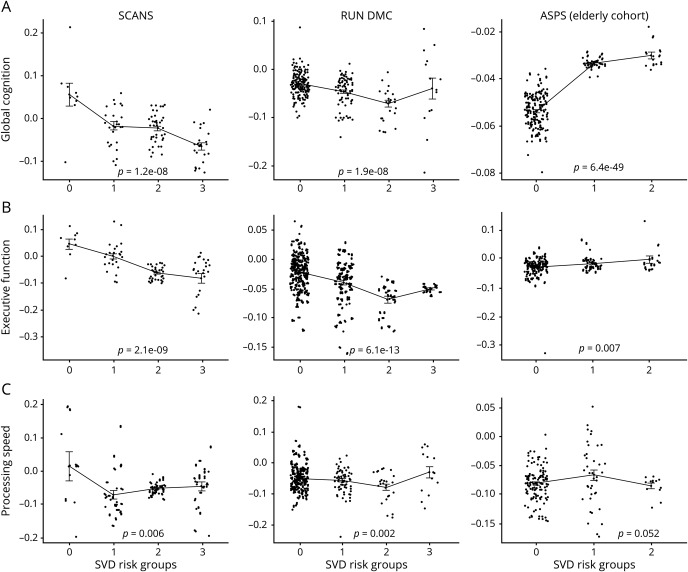
Cognitive change plotted against simple small vessel disease (SVD) score for the 3 cognitive indices (upper panel, global cognition; middle panel, executive function; lower panel, processing speed) for the 3 cohorts (St George's Cognition and Neuroimaging in Stroke [SCANS], Radboud University Nijmegen Diffusion Imaging and Magnetic Resonance Imaging Cohort [RUN DMC], and Austrian Stroke Prevention Study [ASPS]) The slopes were used from a linear mixed effect analysis.

Participants with higher SVD scores showed a greater rate of decline in global cognition and EF compared to participants with lower SVD scores in both SCANS and RUN DMC, and a similar relationship was observed in ASPS. The mean slope differences among SVD groups were statistically significant in SCANS and RUN DMC (table e-3, doi.org/10.17863/CAM.48785 and figure 2). In RUN DMC, there was a similar pattern for PS, but this relationship was not seen in SCANS and ASPS.

### Selection of patients with SVD and its effect on reduction of sample size for a clinical trial

We determined the effect of selecting patients with a higher SVD score (≥2) on sample size for a clinical trial assuming 80% power and 5% type 1 error (see Methods). As there were few cases with high SVD scores in ASPS, this analysis was limited to data from RUN DMC and SCANS. Results are shown in table 5. For dementia onset as the outcome measure, preselection of patients with higher SVD score reduced sample size by 57% in the pooled dataset. For cognitive decline as the outcome measure, sample sizes were reduced by 56%–66% in SCANS and 40%–63% in RUN DMC depending on which cognitive measure (global cognition, EF, or PS) was the primary endpoint.

**Table 5:**
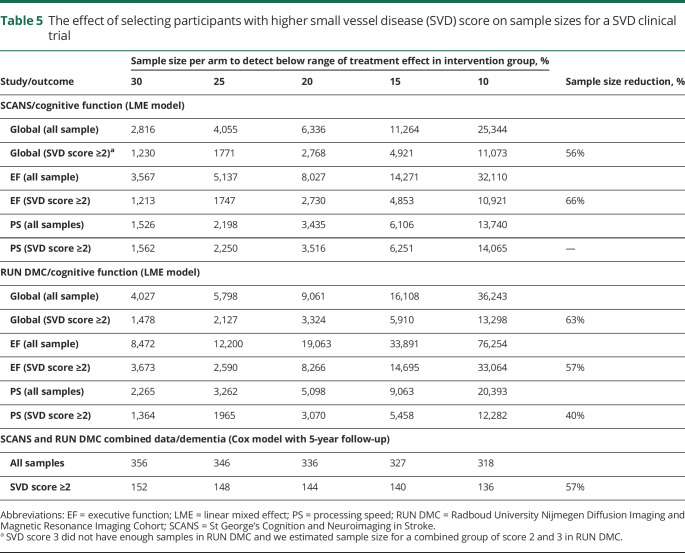
The effect of selecting participants with higher small vessel disease (SVD) score on sample sizes for a SVD clinical trial

## Discussion

In this analysis of 3 longitudinal studies covering a range of SVD severity, we found that a simple SVD score, using measures readily available by simple review of routine clinical MRI scans, can aid in predicting which SVD cases will progress to dementia.

Previous cross-studies^3,4^ have shown such a score associated with cognition and we confirmed this in our analysis of the baseline data. However, whether it can predict dementia can only be determined by using longitudinal data. By following up patients over time in longitudinal prospective cohorts, we were able to demonstrate that the MRI score improved prediction over that provided by clinical markers such as age, sex, and number of years of education. We also found the score could predict cognitive decline consistent with a recent report of it predicting EF in hypertensive individuals.^17^ Our results are consistent with a recent longitudinal study showing a similar MRI summary score predicted dementia in a population-based Swedish cohort.^18^

Whereas the results were broadly consistent across populations, the score appeared more predictive in studies with patients with more severe SVD, defined in SCANS as the presence of confluent WMH. There are several possible explanations for this. First, the range of MRI scores in ASPS was low, with only a small number of patients having higher scores. Second, in cohorts with symptomatic SVD, most cases of dementia are likely to have a vascular basis and therefore to be directly related to the SVD. In contrast, in population-based cohorts such as ASPS, many cases of dementia will be due to nonvascular causes such as AD, to which SVD will make a lesser contribution. A clinical implication is that the MRI score will be most useful in patients who already have significant SVD, although in view of the association between WMH and AD it would be interesting to formally test its predictive value in this population.

To construct the SVD score, a point was given for the presence or absence of MRI features. However, previous studies have shown that not only the presence or absence of lacunar infarcts^15^ and WMH predicts dementia, but the number of lacunar infarcts and the severity of WMH have additional predictive value.^15,19^ Therefore we constructed an amended score in which there were a number of categories for both the number of lacunar infarcts and the severity of WMH. This amended score appeared to improve the predictive ability slightly. SCANS included patients with more severe SVD with a wider range of values; therefore the amended score improved prediction in SCANS slightly more than in the other cohorts. In contrast, ASPS and RUN DMC, with patients with milder SVD, had little prediction improvement. We did not include PVS in the score, as this information was not available in RUN DMC. However, a sensitivity analysis in those 2 populations with PVS showed no added predictive value, consistent with a recent meta-analysis showing no association between PVS and cognition.^20^

We also examined the hypothesis that SVD score may help to identify an enriched group of patients and improve the power of clinical trials. This is of particular importance as studies including patients with all grades of SVD have failed to detect cognitive change during follow-up.^21^ We assessed this for 2 primary endpoints of cognition and dementia and showed that selecting patients with severe SVD (as assessed by simple SVD score) could reduce the sample sizes moderately.

The strengths of this study are that we determined whether SVD scores could predict progression to dementia in longitudinal studies. Furthermore, we replicated results across multiple cohorts and included cohorts with differing severities of SVD. This allowed us to determine whether the predictive value differed based on severity of SVD.

The study has limitations. Although these studies were designed to predict which factor on MRI predicted cognitive decline, they were not primarily created to answer the current clinical question. The analysis involved pooling data from a number of different populations with different follow-up periods and methods of assessing dementia incidence.

A simple MRI SVD score based on visual rating can predict future risk of dementia and cognitive decline.

## Glossary

AD: Alzheimer disease
AIC: Akaike information criterion
ASPS: Austrian Stroke Prevention Study
AUC: area under the curve
CI: confidence interval
CMB: cerebral microbleeds
DSM-IV: Diagnostic and Statistical Manual of Mental Disorders, 4th edition
DSM-5: Diagnostic and Statistical Manual of Mental Disorders, 5th edition
EF: executive function
FLAIR: fluid-attenuated inversion recovery
IQR: interquartile range
LME: linear mixed effect
PS: processing speed
PVS: perivascular spaces
RUN DMC: Radboud University Nijmegen Diffusion Imaging and Magnetic Resonance Imaging Cohort
SCANS: St George's Cognition and Neuroimaging in Stroke
SVD: small vessel disease
WMH: white matter hyperintensities
